# Polymorphisms in the *MBL2* gene are associated with the plasma levels of MBL and the cytokines IL-6 and TNF-α in severe COVID-19

**DOI:** 10.3389/fimmu.2023.1151058

**Published:** 2023-04-17

**Authors:** Maria Alice Freitas Queiroz, Angélica Menezes Santiago, Wandrey Roberto dos Santos Brito, Keise Adrielle Santos Pereira, William Botelho de Brito, Maria Karoliny da Silva Torres, Jeferson da Costa Lopes, Erika Ferreira dos Santos, Flávia Póvoa da Costa, Kevin Matheus Lima de Sarges, Marcos Henrique Damasceno Cantanhede, Mioni Thieli Figueiredo Magalhães de Brito, Andréa Luciana Soares da Silva, Mauro de Meira Leite, Maria de Nazaré do Socorro de Almeida Viana, Fabíola Brasil Barbosa Rodrigues, Rosilene da Silva, Giselle Maria Rachid Viana, Tânia do Socorro Souza Chaves, Adriana de Oliveira Lameira Veríssimo, Mayara da Silva Carvalho, Daniele Freitas Henriques, Carla Pinheiro dos Santos, Juliana Abreu Lima Nunes, Iran Barros Costa, Ednelza da Silva Graça Amoras, Sandra Souza Lima, Izaura Maria Vieira Cayres-Vallinoto, Igor Brasil-Costa, Juarez Antônio Simões Quaresma, Luiz Fábio Magno Falcão, Eduardo José Melo dos Santos, Antonio Carlos Rosário Vallinoto

**Affiliations:** ^1^ Laboratory of Virology, Institute of Biological Sciences, Federal University of Pará (UFPA), Belém, Brazil; ^2^ Graduate Program in Biology of Infectious and Parasitic Agents, Institute of Biological Sciences, Federal University of Pará (UFPA), Belém, Brazil; ^3^ Graduate Program in Virology, Evandro Chagas Institute, Department of Science, Technology, Innovation and Strategic Health Inputs, Ministry of Health of Brazil, Ananindeua, Brazil; ^4^ Laboratory of Genetics of Complex Diseases, Institute of Biological Sciences, Federal University of Pará, Belém, Brazil; ^5^ Laboratory of Basic Research in Malaria, Parasitology Section, Evandro Chagas Institute, Health and Environment Surveillance Secretariat, Brazilian Ministry of Health, Ananindeua, Brazil; ^6^ School of Medicine, Institute of Medical Sciences, Federal University of Pará, Pará, Brazil; ^7^ Belém Adventist Hospital, Belém, Belém, Brazil; ^8^ Laboratory of Immunology, Section of Virology, Instituto Evandro Chagas, Health and Environment Arbovirology and Hemorrhagic Fevers Section, Evandro Chagas Institute, Health and Environment Surveillance Secretariat, Ananindeua, Brazil; ^9^ Laboratory of Immunology, Section of Virology, Instituto Evandro Chagas, Health and Environment Surveillance Secretariat, Brazilian Ministry of Health, Brazilian Ministry of Health, Ananindeua, Brazil; ^10^ Center of Biological and Health Sciences, University of the State of Pará, Belém, Brazil

**Keywords:** COVID-19, long COVID, MBL, polymorphisms, cytokines

## Abstract

**Introduction:**

Mannose-binding lectin (MBL) promotes opsonization, favoring phagocytosis and activation of the complement system in response to different microorganisms, and may influence the synthesis of inflammatory cytokines. This study investigated the association of MBL2 gene polymorphisms with the plasma levels of MBL and inflammatory cytokines in COVID-19.

**Methods:**

Blood samples from 385 individuals (208 with acute COVID-19 and 117 post-COVID-19) were subjected to real-time PCR genotyping. Plasma measurements of MBL and cytokines were performed by enzyme-linked immunosorbent assay and flow cytometry, respectively.

**Results:**

The frequencies of the polymorphic MBL2 genotype (OO) and allele (O) were higher in patients with severe COVID-19 (p< 0.05). The polymorphic genotypes (AO and OO) were associated with lower MBL levels (p< 0.05). IL-6 and TNF-α were higher in patients with low MBL and severe COVID-19 (p< 0.05). No association of polymorphisms, MBL levels, or cytokine levels with long COVID was observed.

**Discussion:**

The results suggest that, besides MBL2 polymorphisms promoting a reduction in MBL levels and therefore in its function, they may also contribute to the development of a more intense inflammatory process responsible for the severity of COVID-19.

## Introduction

Severe acute respiratory syndrome coronavirus 2 (SARS-CoV-2) is responsible for the development of different clinical manifestations of coronavirus disease 19 (COVID-19), which clinically presents as mild, moderate, or severe. The symptoms of acute COVID-19 can range from mild (for example, fever, cough and sore throat) to the most severe (severe pneumonia, acute respiratory distress syndrome – ARDS and multiple organ failure), which are mainly responsible for cases of death from the disease (1). The severity of acute disease is related to several factors, especially age, sex, comorbidities, and dysregulation of the immune-inflammatory response of the host, which can promote intense production of cytokines and trigger immunopathological reactions (2–5).

After recovery from acute COVID-19, some individuals continue to present certain symptoms that were initially related to the phenomenon called “medical gaslighting” (6). However, the emergence of several patients who recovered from acute COVID-19 but who continued to present a variety of symptoms not explained by other causes allowed the identification of a new condition related to infection by SARS-CoV-2, called long COVID syndrome (or post-COVID syndrome), including dyspnea, abdominal pain, diabetes mellitus, myalgia, alopecia, insomnia, anxiety, among others (7–9), which seems to be associated with a persistent inflammatory response (10).

An effective anti-SARS-CoV-2 immune response requires adequate activation of the innate and adaptive immune systems (11). The innate immune response is the body’s first line of defense, playing a key role in viral detection and control (12). Variations in the intensity of some components of the innate immune system are associated with the severity of COVID-19, including the activation of NLR family pyrin domain containing protein 3 (NLRP3), which induces pyroptotic death (13), and the suppression of interferon I (IFN-I) and antiviral activity (14).

The innate immune system is also composed of important soluble mediators that modulate the course of the disease, inducing the release of many innate immune cells into the circulation and their recruitment to infected and inflamed tissues (11). Some soluble molecules act as pattern recognition receptors. Among them, mannose-binding lectin (MBL) is one of the pattern recognition receptors that has strong binding ability to microorganisms, contributing to their opsonization and phagocytosis and the activation of the complement system *via* lectins (15). MBL may also influence the immune response by modulating the levels of proinflammatory cytokines. Low MBL has been associated with the production of high levels of several cytokines, mainly interleukin 6 (IL-6) and tumor necrosis factor alpha (TNF-α) (16–19).

MBL is one of the recognition molecules of the lectin pathway that binds to the S and N proteins of SARS-CoV-2, after which the virus induces the activation and deposition of C3b and C4b, which demonstrates a significant participation of lectins in the immunopathogenesis of COVID-19 (20). The participation of MBL in opsonization seems to be necessary for the neutralization of SARS-CoV-2 (21).

Single nucleotide polymorphisms (SNP) have been described in exon 1 of the MBL2 gene, and identified from the reference SNP (rs) number: rs1800450 (MBL2* B), rs1800451 (MBL2* C) and rs5030737 (MBL2* D), and collectively termed the *O allele, which promote changes in the protein structure leading to a reduction in serum levels of functional MBL. Variant alleles that cause low plasma concentrations of functional MBL have been associated with an increased risk for the development of infections (22–25).

The present study evaluated the frequency of exon 1 polymorphisms of the *MBL2* gene in patients with severe COVID-19 and patients with long COVID. It also investigated the association of plasma MBL level, inflammatory cytokine levels, and *MBL2* polymorphisms with the severity of COVID-19 infection and with the development of long COVID.

## Materials and methods

### Sample characterization and collection

The present study included blood samples from 385 individuals investigated for acute COVID-19 (n= 208) and long COVID (n= 177). Of the 208 patients with acute COVID-19, 71 were characterized by a severe clinical condition, and 137 were characterized by a nonsevere clinical condition (mild to moderate). The classification was performed according to the criteria established by the World Health Organization (1). Patients with acute COVID-19 had blood samples collected with a medium of 6.5 days after the diagnosis of infection.

Of the patients evaluated in the post-COVID period, 111 had a clinical diagnosis of long COVID (post-COVID syndrome), and 66 people did not have long COVID. The diagnosis of long COVID was based on the presence of symptoms that persisted for at least 3 months after resolution of the infection and that had no specific cause and/or relationship with other morbidities. People in the group without long COVID were followed up for 6 months after resolution of the acute infection to confirm the absence of COVID-related symptoms. **Table 1** describes the main symptoms of individuals with severe COVID-19, non-severe COVID-19 and long COVID.

**Table 1:** Main symptoms related to patients with different clinical conditions of COVID-19.

Clinical condition	Symptoms
Non-severe COVID-19	Fever, cough, fatigue, anorexia, sore throat, nasal congestion, headache, diarrhoea, nausea and vomiting and anosmia; Some patients had rapid breathing and mild pneumonia.
Severe COVID-19	Severe pneumonia; Chest imaging (radiograph, CT scan or lung ultrasound: bilateral opacities); Impairment of oxygenation. All patients were admitted to hospitals for treatment of complications of the disease.
Long COVID	Dyspnea, chest pain, muscle weakness, tremor, fatigue, myalgia, headache, visual changes, insomnia.

The evaluation included individuals of both sexes, aged 18 years or older, not vaccinated against SARS-CoV-2, treated at the COVID-19 outpatient clinic of the University of the State of Pará, Hospital Adventista de Belém, or Instituto Evandro Chagas from July 2020 to May 2021. The post-COVID-19 patient group included those who sought the long COVID outpatient clinic of the University of the State of Pará. Until May 2021, a part of the population had already received a dose of vaccine against COVID-19, however, these individuals were excluded from the study.

Blood samples (10 mL) were collected by venipuncture using a vacuum collection system containing ethylenediaminetetraacetic acid as an anticoagulant. The samples were transported to the Laboratory of Virology, Federal University of Pará, where they were processed for separation of plasma and leukocytes. Leukocyte samples were used for DNA extraction, and plasma samples were used for the measurement of MBL and cytokines.

### DNA extraction

DNA was extracted from peripheral blood leukocytes using a Puregene™ kit (Gentra Systems, Inc., Minneapolis, Minnesota, USA) according to the manufacturer’s protocol, which included the steps of cell lysis, protein precipitation, and precipitation and hydration of the DNA. After extraction, the DNA obtained was quantified by spectrophotometric reading in BioDrop™ equipment (Bio-Rad, Hercules, California, USA) following the protocol recommended by the manufacturer.

### Genotyping of *MBL2* rs1800450 (MBL2* B), rs1800451 (MBL2* C) and rs5030737 (MBL2* D)

Genotyping was performed by real-time PCR using the StepOnePLUS™ Real-Time PCR System (Thermo Fisher, Carlsbad, California, USA). The reactions consisted of the commercially obtained TaqMan™ assays *MBL2* rs1800450 (C:2336609_20), rs1800451 (C:2336610_20), and rs5030737 (C:2336610_10), containing specific primers and probes for amplification of the target sequence (Thermo Fisher, Carlsbad, California, USA). The reaction consisted of 1x MasterMix, H_2_O, 20x assay C_11537906_20, and 50 ng DNA. The cycling program was 10 minutes at 95°C and 40 cycles of 15 seconds at 95°C and 1 minute at 60°C.

The wild-type and polymorphic alleles (MBL2* A, *B, *C, and *D) were evaluated based on genotypic clustering, with genotypes AA and OO formed by the homozygous combination of wild-type and polymorphic alleles at the three loci in exon 1, respectively.

### Plasma MBL measurement

MBL was quantified using the Invitrogen Human MBL ELISA Kit (ThermoFisher, Carlsbad, CA, USA), which uses specific monoclonal antibodies to detect the protein. The test was performed according to the manufacturer’s instructions.

### Plasma measurement of cytokines

Cytokine levels were quantified by flow cytometry using the Human Th1/Th2/Th17 Cytometric Bead Array Kit (CBA) (BD Biosciences, San Diego, CA, USA) in BD FACS Canto II equipment. All procedures followed the manufacturer’s guidelines. The method used was based on beads conjugated with the capture antibody, in which six populations of beads with different fluorescence intensities conjugated to a capture antibody specific for each cytokine were mixed to form CBA and then read in FL channel 3 on the flow cytometer.

### Statistical analysis

The information obtained was entered into a database using Microsoft Office Excel 2013 software. The allelic and genotypic frequencies of the polymorphisms were determined by direct counting, and the differences between groups were evaluated by the chi-square test, odds ratio (OR) test, Fisher’s exact test, and G test. The normality of the distribution of MBL and cytokine levels was analyzed with the Shapiro–Wilk test. Given the results of the normality test, the differences in the plasma levels of these markers between groups were evaluated using the nonparametric Mann–Whitney test, and the levels of the markers between the MBL haplotypes were compared using the Kruskal–Wallis test. Correlations between MBL and cytokine levels were calculated as Spearman’s correlation coefficient. The tests were performed using the programs BioEstat 5.3 and GraphPad Prism 5.0, and associations with p < 0.05 were considered significant.

## Results

The genotyping of the polymorphisms in the *MBL2* gene in the investigated groups showed the presence of the homozygous BB and CC genotypes, but the DD genotype was not detected. In the group with acute COVID-19, the genotype distribution differed between the two clinical subgroups. Subjects with severe COVID-19 had a significantly higher proportion of the homozygous polymorphic genotype (OO) than non-severe subjects (15.49% *vs* 4.37%; p= 0.0207). Residue analysis indicated that the difference in genotypic frequencies between the groups was related to the higher frequency of the homozygous polymorphic genotype (OO) in the group with severe COVID-19. Patients with the polymorphic genotype (OO) had a higher chance (OR= 3.89) of developing severe disease than those homozygous for the wild-type genotype (AA). The comparison of disease frequencies between AO heterozygotes and AA homozygotes did not show statistical significance (OR= 0.92; CI= 0.48-0.93; *p*= 0.9391). The polymorphic O allele was more frequent in the group of patients with severe COVID-19 (*p*= 0.0274; OR= 1.74) (**Table 2**).

**Table 2:** Evaluation of the frequencies of polymorphisms in the *MBL2* gene between patients with severe and non-severe manifestations of acute COVID-19.

*MBL2* genotypes	Severe COVID-19 n = 71 n (%)	Non-severe COVID-19 n = 137 n (%)	*p*	OR (95% CI)	
AA	40 (56.34)	85 (62.04)	0.0207^†^		
AO	20 (28.17)	46 (33.58)		3.89 (1.34-11.28)^a^	
OO	11 (15.49)^+^	6 (4.37)		*p* = 0.0179	
*A	0.70	0.79	0.0272	1.74 (1.74-2.79)^b^
*O	0.30	0.21		*p* = 0.0272
*BB*	6 (8.45)	4 (2.92)	0.0893^#^	-
*CC*	5 (7.04)	2 (1.45)	0.0452#	-

n, number of individuals; *allele; ^†^ chi-squared test; ^+^ residue analysis; ^a^ homozygous polymorphic vs. wild-type homozygote; ^b^ allele O vs. allele A; ^#^ Fisher’s exact test (wild-type homozygote vs. polymorphic homozygote).

The evaluation of genotypes and polymorphic alleles in the *MBL2* gene among individuals in the post-COVID period showed that there were no differences in frequencies between the groups with and without long COVID (**Table 3**).

**Table 3:** Frequencies of polymorphisms in the *MBL2* gene in the groups of individuals with and without long COVID.

*MBL2* genotypes	Long COVID n = 111 n (%)	No long COVID n = 66 n (%)	*p*
AA	75 (67.58)	35 (53.03)	0.1021^†^
AO	28 (25.22)	27 (40.91)	
OO	8 (7.20)	4 (6.06)	
*A	0.80	0.79	
*O	0.20	0.21	
*BB*	5 (4.50)	2 (3.03)	0.6111^#^
*CC*	3 (2.70)	2 (3.03)	0.5157^#^

n: number of individuals; *allele; ^†^ G test. ^#^ Fisher’s exact test (wild-type homozygote vs. polymorphic homozygote).

**Figure 1:**
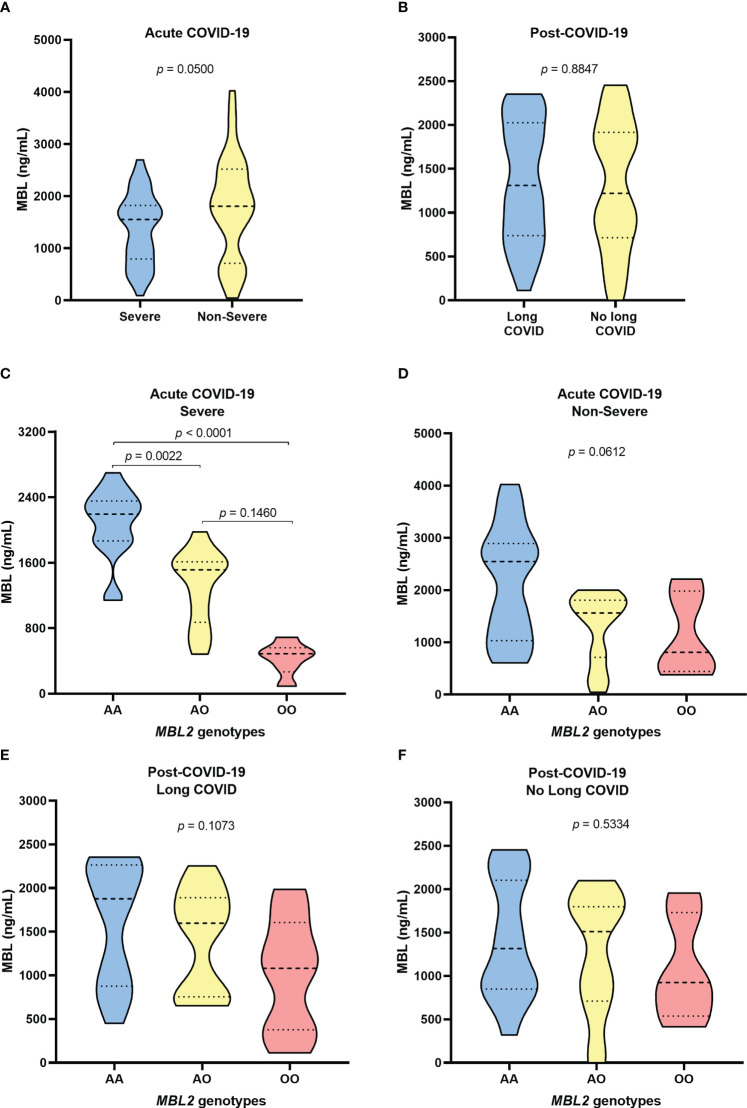
Evaluation of MBL levels among **(A)** patients with severe and non-severe acute COVID-19; **(B)** individuals with long COVID and individuals without the syndrome in the post-COVID period; carriers of the different *MBL2* genotypes with **(C)** severe and non-severe **(D)** acute COVID; and carriers of the different *MBL* genotypes **(E)** with and **(F)** without long COVID.

The measurement of plasma MBL levels showed that patients with severe acute COVID-19 had lower MBL than individuals with a non-severe diagnosis (*p*= 0.0500; **Figure 1A**). In the post-COVID evaluation, MBL levels were not different between the groups with and without long COVID (*p*= 0.8847; **Figure 1B**).

Among the *MBL2* genotypes, the protein levels were significantly lower in individuals with heterozygous and homozygous polymorphic genotypes than in those with the wild-type genotype (*p*= 0.0022 and *p*< 0.0001, respectively; **Figure 1C**). Individuals with non-severe clinical manifestations with polymorphic genotypes also had lower levels of MBL, but the difference was not statistically significant (*p*> 0.05; **Figure 1D**).

In the evaluation of the post-COVID-19 group, patients with long COVID who carried the polymorphic genotypes had lower MBL levels than those with the AA genotype, but the differences were not significant (*p*> 0.05; **Figure 1E**). Individuals who did not develop symptoms of long COVID showed no differences in MBL levels between genotypes (*p*> 0.05; **Figure 1F**).

**Figure 2:**
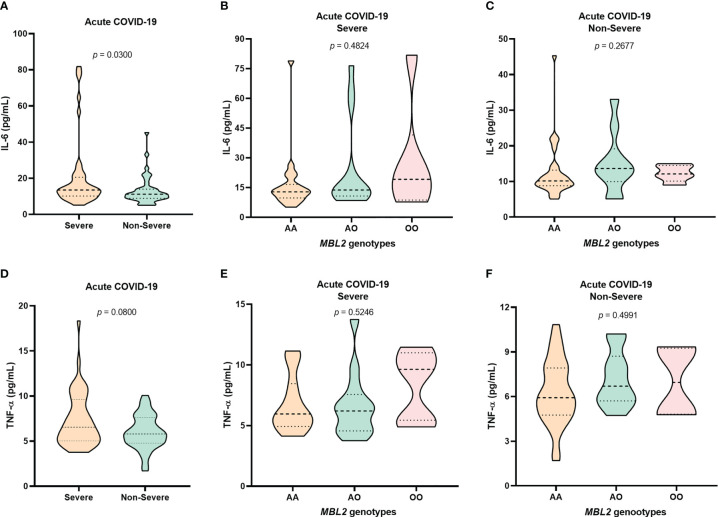
Evaluation of IL-6 levels among **(A)** patients with severe and non-severe acute COVID-19 and among carriers of the different *MBL2* genotypes with **(B)** severe and **(C)** non-severe forms of the disease. TNF-α levels among **(D)** patients with severe and non-severe acute COVID-19 and among carriers of the different *MBL2* genotypes with **(E)** severe and **(F)** non-severe forms of the disease.

As the variations in MBL levels were greater in the group of patients with severe acute COVID-19, the levels of the cytokines IL-6 and TNF-α were evaluated in relation to this form of the disease. The levels of IL-6 (*p*= 0.0300; **Figure 2A**) and TNF-α (*p* = 0.0800; **Figure 2D**) were higher in patients with severe COVID-19 than in those with the non-severe form. Regarding the genotypic characterization of *MBL2*, patients with polymorphic genotypes had higher levels of IL-6 (**Figures 2B, C**) and TNF-α (**Figures 2E, F**), but without statistical significance (*p*> 0.05). The levels of the cytokines IL-17, IFN-γ, IL-10, IL-4, and IL-2 showed no significant differences between *MBL2* genotypes.

**Figure 3:**
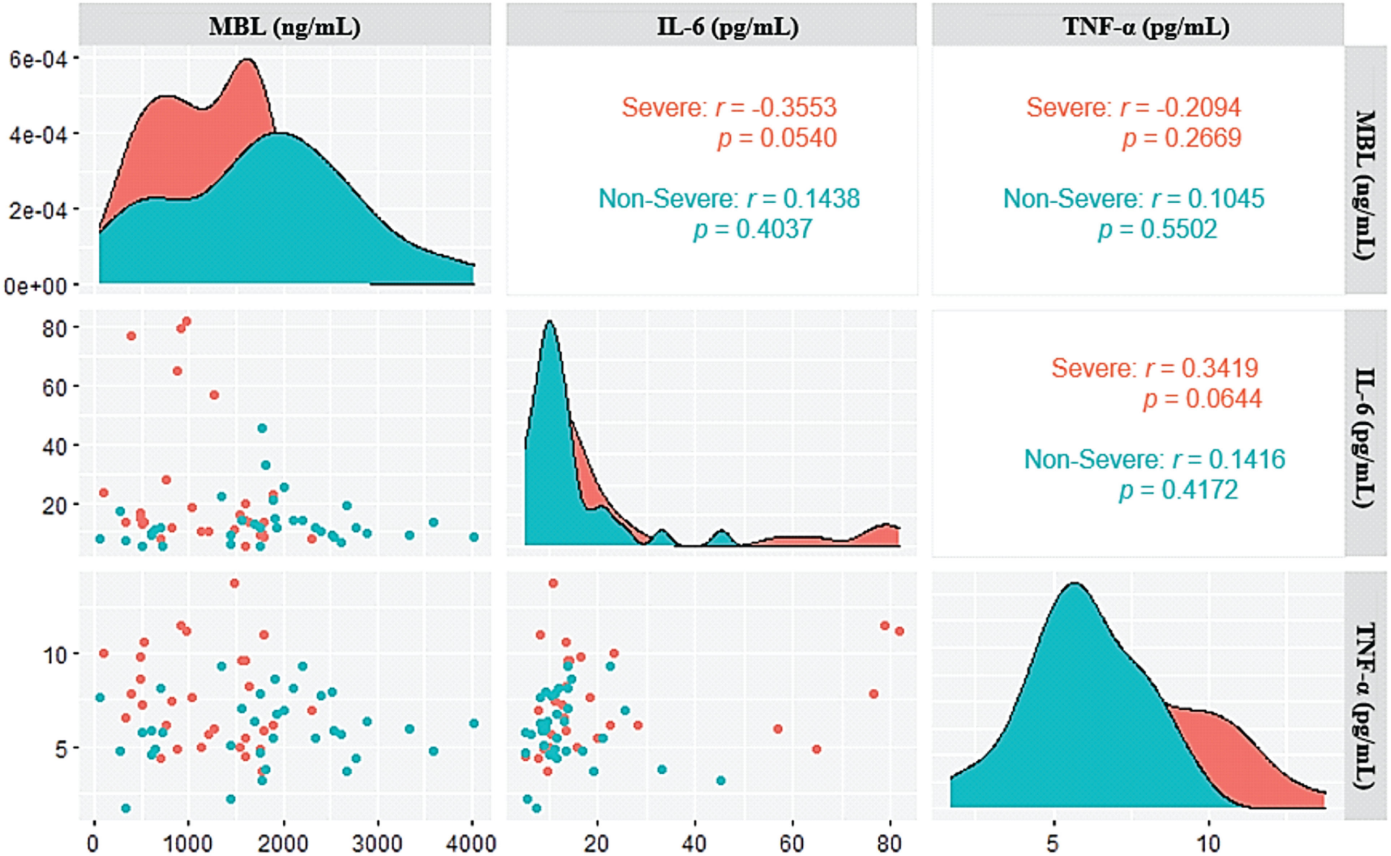
Correlogram of the plasma levels of MBL, IL-6, and TNF-α in patients with severe and non-severe acute COVID-19.

The correlation analysis between the plasma levels of MBL and the cytokines IL-6 and TNF-α showed a trend toward a negative correlation between the levels of MBL and IL-6 (*r*= -0.3553; *p*= 0.0540) and a trend toward a positive correlation between IL-6 and TNF-α levels (*r*= 0.3419; *p*= 0.0644) in the group with severe COVID-19 (**Figure 3**).

## Discussion

COVID-19 is a complex infectious disease whose severity involves several risk factors such as genetic characteristics of the host, which may influence the development of a competent immune response against SARS-CoV-2 (26–28). Another worrying aspect of the disease has been the presence of symptoms related to long COVID. These symptoms can occur in different amounts and types of clinical manifestations, including cognitive and mental impairments, chest and joint pains, palpitations, myalgia, smell and taste dysfunctions, cough, headache, and gastrointestinal and cardiac problems (9).

The present study investigated the association of *MBL2* genotypes with the severity of acute COVID-19 and long COVID. The results showed that the homozygous polymorphic genotype (OO) was more prevalent in the group of patients with severe COVID-19 than those patients who developed the mildest manifestations of the disease. In contrast, no association of the polymorphic genotype with long COVID was observed.

The polymorphic genotypes of the *MBL2* gene have been associated with the severity and progression of different viral diseases, including HTLV-1 (29), HIV-1 (30), and hepatitis B (31), hepatitis C (32), and dengue (33). Some studies that evaluated polymorphisms in the *MBL2* gene in groups of patients with severe and non-severe clinical forms of COVID-19 observed association of variant B (rs1800450) with the severity of the disease. Medetalibeyoglu et al. evaluated the polymorphisms in COVID-19 patients from Turkey and observed that the polymorphic genotype BB, associated with a deficient protein, was related to the acquisition of COVID-19 and the severe clinical course of the disease, demonstrated by the greater risk of developing severe symptoms and the need for ICU admission (34). A study that evaluated the presence of exon 1 polymorphisms in patients of different ethnicities (Greek, Turkish, Ukrainian, Indonesian, Uzbek, Moldovan, American, and Cuban) found an association of the polymorphism rs1800450 with the severity of COVID-19 (35). Although these studies were conducted in mostly Eastern European populations, unlike the population investigated in the present study, which was of mixed race, with genetic contributions of whites, blacks, and indigenous people (36), the association of polymorphisms in the *MBL* 2 gene with the severity of COVID-19 suggests that the genetic influence of these variations seems to be independent of ethnic differences.

However, studies conducted in Italy showed that other different polymorphisms in the *MBL2* gene were associated with susceptibility to SARS-COV-2 infection (37, 38). Hultström et al. demonstrated that some *MBL2* haplotypes, which had reduced protein activity, were related to the risk of thromboembolic complication in critical patients with disease (39). These studies show that not only the missense *MBL2* gene variants (B, C and D) can influence COVID-19, other polymorphisms are relevant and should also be evaluated in the disease in other different populations.

These data show that polymorphisms in exon 1 of the *MBL2* gene, associated with reduced protein levels, may be a genetic factor that promotes a significant contribution to the dysregulation of innate immune control and leads to the development of more severe manifestations of COVID-19. Most likely, polymorphic genotypes induce the most severe manifestations because they promote a reduction in circulating levels of MBL and thereby lower the activation of the complement system, opsonization, and phagocytosis (15). The activation of these mechanisms is important in reducing viral load levels and eliminating the infection.

Long COVID is a condition that is still not well understood, as it has not yet been possible to establish why symptoms persist in some individuals. The lack of association between MBL genotypes and long COVID shows that the manifestations of this syndrome are not related to genetic variations that influence MBL expression. Future studies should identify whether the symptoms of long COVID are due to an altered and persistent immune response, if polymorphisms in the genes of the immune-inflammatory response can influence its evolution, or if long COVID results from damage caused by the virus to specific tissues.

The evaluation of plasma MBL levels showed that patients with the severe form of COVID-19 during acute infection had lower levels of the protein than those with the non-severe form of the disease. Other studies that evaluated polymorphisms in MBL genes between patients with severe COVID-19 and patients with non-severe forms did not quantify MBL plasma levels (34, 35). On the other hand, studies that investigated MBL plasma levels in COVID-19, evaluated only patients admitted to hospitals and patients with critical disease, in these studies it was possible to observe association of MBL levels with worst prognosis of the disease (37, 39).

As MBL performs important functions against infectious agents, which help in the elimination of infections, the reduction in the circulating level of MBL may facilitate the persistence of several pathogens in the body (40, 41). The low levels of MBL observed in patients with the severe form of acute COVID-19 may suggest a deficiency in MBL functions, resulting in greater replication of the virus and the involvement of various tissues, which are the main characteristics of the severe form of the disease.

Previous studies demonstrated association of MBL plasma levels with acute COVID-19 MBL2 gene polymorphisms, where patients with polymorphic genotypes had lower levels of MBL (37, 39), but these assessments were made only in patients with severe COVID-19 forms. In our results it was possible to observe variations in plasma MBL levels in relation to polymorphic genotypes in patients with severe COVID-19 manifestations and patients with milder symptoms of the disease and showed that in both groups, polymorphic genotypes were associated with lower levels of MBL, but in the group of patients with severe COVID-19 this reduction was much more significant. The low levels of MBL observed in patients with the severe form of COVID-19 seem to be influenced by the polymorphic genotypes that had lower levels of MBL and were more frequent in this group. In contrast, polymorphisms in the MBL gene were not associated with long COVID. Because this condition is characterized by clinical manifestations that persist for at least 3 months after resolution of the acute disease, the results obtained in this study suggest that the polymorphic genotypes evaluated are associated with disease severity in the acute phase, but after resolution of the infection, the polymorphisms do not influence the onset and persistence of other symptoms.

Because low levels of MBL seem to influence the production of proinflammatory cytokines, especially IL-6 and TNF-α (16–19), the present study evaluated the levels of these cytokines in patients in the acute phase of COVID-19 and observed that patients with the severe form of the disease who had lower levels of MBL had higher levels of IL-6 and TNF-α, with a slight negative correlation between MBL and IL-6 levels and a positive correlation between IL-6 and TNF-α levels. The cytokine levels were higher in patients with polymorphic MBL genotypes and patients who had lower plasma levels of the protein compared to those with the wild-type genotype, but the differences were not significant.

In natural killer cells, MBL suppresses the levels of the cytokines TNF-α and IFN-γ as well as surface activation markers, such as CD25 and CD69, that are responsible for the activation of different cells of the innate and adaptive immune response (16). High concentrations of MBL drastically reduced IL-6 and TNF-α production by monocytes in response to meningococcal infection, and lower concentrations increased IL-6 production, suggesting that in addition to MBL being involved in complement activation, it is a potent regulator of the inflammatory response and may affect the severity of infectious diseases (17).

The proinflammatory activity of IL-6 plays an important role in the immune response. In COVID-19, high levels of IL-6 have been associated with the severe form of the disease and worse prognosis of infection (10, 42, 43). The results of the present study suggest that low MBL levels may contribute to the increase in IL-6 in patients with severe COVID-19. These mechanisms seem to be related to the reduction in the activation of Toll-like receptors (TLRs). Liu et al. demonstrated that high MBL levels promote its interaction with poly(I:C), a sdRNA, and suppress the activation of TLR3 pathways and the subsequent production of cytokines (19). MBL has also been associated with suppression of TLR4 and TLR9 activity and reduction of NF-κB activation (17, 44). Thus, in addition to critically ill patients presenting an impairment in the opsonization and phagocytosis of SARS-CoV-2, these patients develop a more pronounced inflammatory process, which results in greater damage to the affected tissues.

MBL plays a central role in inflammation, coagulation, and immunity and is a central point of intersection for these systems. Therefore, the coordinated production of this protein contributes to maintaining blood fluidity and preserving homeostasis throughout the body. Experimental studies in animal models show that in viral infections, MBL deficiency can lead to a delayed immune response in the lungs and predispose patients to more serious clinical conditions, including disseminated intravascular coagulation and multiple-organ damage (40). These are characteristic manifestations of the severe form of COVID-19, which suggests that polymorphisms in the *MBL2* gene that lead to MBL deficiency may significantly contribute to the systemic dysregulation of the organism.

Differences in MBL levels were not associated with long COVID. Although some studies have evaluated the inflammatory response in long COVID, only certain symptoms of the syndrome seem to be related to a persistent inflammatory response (45–47). As this is the first work that investigated MBL levels in long COVID, these initial results suggest that MBL functions do not contribute to the development of symptoms in the post-COVID-19 period.

Although the study has relevant information for a better understanding of how genetic variations in immune response components may be associated with the severity of COVID-19, it has some limitations, mainly related to the lack of evaluation of other polymorphisms in the *MBL2* gene and the expression of immune components that induce the production of TNF-α and IL-6 cytokines such as TLR-3 and TLR-7.

In summary, the present study showed that the frequency of polymorphic genotypes of exon 1 of the *MBL2* gene was associated with the severe form of acute COVID-19 and with reduced plasma levels of MBL. In addition, patients with the severe form of the disease who had low levels of MBL had higher levels of the inflammatory cytokines IL-6 and TNF-α. These results show that *MBL2* polymorphisms, responsible for promoting a reduction in MBL levels and, therefore, in its function, contribute to the severity of COVID-19 and, low levels of MBL can help to intensify the inflammatory process, characteristic of severe disease.

## Data availability statement

The original contributions presented in the study are included in the article/Supplementary Material. Further inquiries can be directed to the corresponding author.

## Ethics statement

The studies involving human participants were reviewed and approved by National Research Ethics Committee (CAEE: 33470020.1001.0018). The patients/participants provided their written informed consent to participate in this study.

## Author contributions

ACV, LF, IC-V and EdS conceived of the project. MQ, ACV, IBC and EdS wrote and reviewed the manuscript. MQ and SL performed the statistical analyses. MQ, AngS, WRB, KP, WBB, MT, JL, EdS, FC, KS, MC, MB, AS, ML, MV, FR, RS, GV, TC, AOV, MC, DH, CS, JN, IC, EA, SL, JQ, LF, and IB-C collected the biological samples and performed the laboratory analyses. All authors contributed to the article and approved the submitted version.
